# Integrated Analysis of circRNA-miRNA-mRNA ceRNA Network in Cardiac Hypertrophy

**DOI:** 10.3389/fgene.2022.781676

**Published:** 2022-02-08

**Authors:** Yang-Hao Chen, Ling-Feng Zhong, Xia Hong, Qian-Li Zhu, Song-Jie Wang, Ji-Bo Han, Wei-Jian Huang, Bo-Zhi Ye

**Affiliations:** ^1^ The Key Laboratory of Cardiovascular Disease of Wenzhou, Department of Cardiology, The First Affiliated Hospital of WenZhou Medical University, WenZhou, China; ^2^ Coronary Care Unit, The First Affiliated Hospital of Wenzhou Medical University, WenZhou, China; ^3^ Department of Cardiology, The Second Affiliated Hospital of Jiaxing University, Jiaxing, China

**Keywords:** cardiac hypertrophy, competitive endogenous RNA, bioinformatics, circRNA, miRNA

## Abstract

Cardiac hypertrophy is an adaptive cardiac response that accommodates the variable hemodynamic demands of the human body during extended periods of preload or afterload increase. In recent years, an increasing number of studies have pointed to a potential connection between myocardial hypertrophy and abnormal expression of non-coding RNAs. Circular RNA (circRNA), as one of the non-coding RNAs, plays an essential role in cardiac hypertrophy. However, few studies have systematically analyzed circRNA-related competing endogenous RNA (ceRNA) regulatory networks associated with cardiac hypertrophy. Therefore, we used public databases from online prediction websites to predict and screen differentially expressed mRNAs and miRNAs and ultimately obtained circRNAs related to cardiac hypertrophy. Based on this result, we went on to establish a circRNAs-related ceRNA regulatory network. This study is the first to establish a circRNA-mediated ceRNA regulatory network associated with myocardial hypertrophy. To verify the results of our analysis, we used PCR to verify the differentially expressed mRNAs and miRNAs in animal myocardial hypertrophy model samples. Our findings suggest that three mRNAs (Col12a1, Thbs1, and Tgfbr3), four miRNAs (miR-20a-5p, miR-27b-3p, miR-342-3p, and miR-378a-3p), and four related circRNAs (circ_0002702, circ_0110609, circ_0013751, and circ_0047959) may play a key role in cardiac hypertrophy.

## Introduction

The main function of the heart is to regulate blood output in response to the changing hemodynamics of the body, which enables the heart to meet the needs of the body under normal and stress conditions. To successfully accomplish this task in the presence of a prolonged increase in preload or afterload, cardiac muscle cells usually undergo a certain volume increase, a condition known as hypertrophy (Nakamura and Sadoshima, 2018). It is well known that although cardiac hypertrophy begins as an adaptive response, chronic cardiac hypertrophy can eventually develop into heart failure and eventual death. Since cardiac hypertrophy is a key risk factor for cardiac failure, there is a pressing need to study its molecular biological mechanism in the progression of cardiac hypertrophy and discover its potential therapeutic targets.

In recent years, mounting evidence shows that the non-coding RNAs play a crucial role in cardiovascular diseases, especially in the process of cardiac hypertrophy (Poller et al., 2018). Non-coding RNA is a type of RNA that lacks protein-coding functions, which can modulate biological processes by regulating the expression of coding RNAs in various ways (Matsui and Corey, 2017). Non-coding RNAs are mainly divided into micro RNA (miRNA), circular RNA (circRNA), long non-coding RNA (lncRNA), and extracellular RNA (exRNA) (Ling et al., 2013; Sato-Kuwabara et al., 2015; St. Laurent et al., 2015; Ebbesen et al., 2016). The interactions between these RNAs eventually form a competing endogenous RNA (ceRNA) regulatory network, a concept proposed by Salmena (Salmena et al., 2011). Different from other non-coding RNAs, circRNAs are not vulnerable to the degradation of RNA enzymes and are more stable than linear RNAs (Jeck et al., 2013). CircRNA has a variety of molecular functions in sponge miRNA, regulatory transcription, regulatory RNA binding proteins, and even coding proteins (Chen et al., 2021). What gets the most attention is that some circRNAs contain miRNA response elements (MREs), making them miRNA sponges that reduce miRNA binding to mRNA (Hansen et al., 2013). To date, circRNAs have been involved in the occurrence and progression of various diseases, including cancer, neurological diseases, and cardiovascular diseases (Lei et al., 2020; Lim et al., 2020; Mehta et al., 2020). In recent years, researchers have begun to pay attention to the role of circRNA in regulating the physiological and pathological processes of cardiac hypertrophy (Li et al., 2020). CircRNA HRCR acts as an endogenous miR-223 sponge, isolating and inhibiting miR-223 activity, resulting in increased ARC expression (Wang et al., 2016). CircRNA Slc8a1 can act as an endogenous sponge for miR-133a in cardiomyocytes (Lim et al., 2019). However, few studies have delved into the mechanism underlying the regulation of cardiac hypertrophy by the circRNA-related ceRNA network.

Therefore, in this study, we downloaded mRNA and miRNA expression data related to cardiac hypertrophy from a public database (Aggarwal et al., 2014). This database included three controls and three samples of myocardial hypertrophy. The researchers of this database performed microarray analysis of mRNA and high-throughput sequencing of miRNA. We used Limma p and DESeq2 packages to screen out differentially expressed mRNAs and miRNAs. Then, we used TarBase and miRTarBase to forecast the targeting miRNAs of the differentially expressed genes (DEGs). Subsequently, the predicted miRNAs and differentially expressed microRNAs (DEMs) were intersected, and then circBank was used to predict the related circRNAs of the miRNAs obtained from the intersection. Finally, a circRNA-related ceRNA network was established. To further obtain the key ceRNA network, we used the Degree module in CytoHubba for analysis and obtained 4 mRNAs, 13 miRNAs, and 5 circRNAs. We then used PCR to detect the expression levels of these mRNAs and miRNAs in animal models of cardiac hypertrophy. Finally, a hub ceRNA network, including three mRNAs, four miRNAs, and four circRNAs, was obtained. In this study, a circRNA-mediated ceRNA regulatory network related to cardiac hypertrophy was established for the first time. This ceRNA regulatory network will provide new data for further elucidation of the mechanism of myocardial hypertrophy and open new avenues of research into therapeutic targets and drug development of cardiac hypertrophy.

## Materials and Methods

### Microarray Data and miRNA Sequencing Data

The microarray data of GSE60291 and the miRNA sequencing data of GSE60292 were obtained from the Gene Expression Omnibus (GEO, http://www.ncbi.nlm.nih.gov/gds/) of the National Center for Biotechnology Information (NCBI). The GSE60291 dataset was based on the GPL570 (Affymetrix Human Genome U133 Plus 2.0 Array) while the GSE60292 dataset was based on the GPL17301 (Ion Torrent PGM). Both databases contain six samples, including three hypertrophy samples and three control samples.

### Data Preprocessing and Identification of Differentially Expressed Genes

To explore the DEGs, we applied the limma package for processing GSE60291 (Ritchie et al., 2015). False positives can be corrected by adjusting the *p* value. The Benjamini–Hochberg method was used to calculate the false discovery rate (FDR), which further improves the reliability of identifying statistically significant genes. We considered an adjusted *p*-value < 0.05 and |log2FC| > 2 as the thresholds for statistical significance. Finally, we used R 4.0.2 (ggplot2 package and pheatmap package) to visualize the differential genes that had been screened out.

### Gene Ontology and Kyoto Encyclopedia of Genes and Genomes Enrichment Analysis of Differentially Expressed Genes

To investigate the biological function of DEGs, the clusterProfiler package was utilized to perform Gene Ontology (GO) and Kyoto Encyclopedia of Genes and Genomes (KEGG) pathway analysis (Yu et al., 2012). We adopted the nominal level of FDR <0.05 to identify statistically significant differences in biological process (BP), cellular component (CC), molecular function (MF), and KEGG analysis. R 4.0.2 was used to visualize the top eight results of the GO term and KEGG pathway enrichment analysis.

### Construction of a Protein–Protein Interaction Network

The protein–protein interaction (PPI) network of DEGs was constructed by the Search Tool for the Retrieval of Interacting Genes (STRING, https://www.string-db.org/) online database (Szklarczyk et al., 2021). We set the minimum required interaction score to 0.4. Other indicators were set as default parameters. The information of nodes and edges was exported as a .txt file and then visualized in Cytoscape 3.8.1.

### Definition of Differentially Expressed MicroRNAs and Interaction of mRNA-miRNA

Subsequently, we applied the DESeq2 package for obtaining the DEMs from GSE60292 as the high-throughput sequencing data conformed to the Poisson distribution (Love et al., 2014). The adjusted *p* < 0.05 and |log2FC| > 1 were used as cut-off values for DEM screening. TarBase v8.0 (http://www.microrna.gr/tarbase) and miRTarBase v8.0 (http://miRTarBase.cuhk.edu.cn/) were used to predict the targeting miRNAs of DEGs (Karagkouni et al., 2018; Huang et al., 20202020). We only retained miRNAs that were present in both downregulated DEMs and the targeting miRNAs of upregulated genes. Similarly, we obtained the miRNAs in the interaction of upregulated DEMs and the targeting miRNAs of downregulated genes.

### Targeting CircRNA of Differentially Expressed MicroRNAs and Construction of Competing Endogenous RNAs Network

To further build the circRNA-related ceRNA network of cardiac hypertrophy, we used Circbank (http://www.circbank.cn/) to predict targeting circRNAs (Liu et al., 2019). We selected circRNAs with good conservation simultaneously. Subsequently, we ranked the circRNAs according to the predicted times. We selected the circRNAs with the top three connectivity and established corresponding ceRNA.

### Definition of Key Competing Endogenous RNAs Network

CytoHubba was used to acquire the key ceRNA network (Chin et al., 2014). According to the degree of nodes, we selected the top 25% nodes in two ceRNA networks, respectively, and only the complete circRNA-miRNA-mRNA axis was retained. Finally, two key ceRNA networks were obtained.

### Animals and Models

All the experimental procedures regarding animal care and laboratory procedures were approved by relevant authorities. Approval of the animal welfare policy was obtained from the Committee on Animal Care of The Second Affiliated Hospital of Jiaxing University (approval document No. JXEY-2019JX097), and it was in line with NIH guidelines (guidelines for the care and use of laboratory animals). Male C57BL/6 wild-type mice (8 weeks old) were obtained from Wenzhou Medical College. All of the mice were housed with a 12 h:12 h light-dark cycle at a constant room temperature and fed a standard rodent diet. The mice adapted for a minimum of 2 weeks before the researchers initiated the experiments.

To establish a mouse model of cardiac hypertrophy induced by angiotensin II (ANGII), we randomly divided the mice into two groups: (I) Ang II group, n = 6 and (II) PBS group, n = 6. For the AngII group, we injected AngII (1.4 mg/kg/day in phosphate buffer, pH 7.2) into the micropump and placed the micropump subcutaneously. The PBS group was prepared in the same way except that PBS was used instead of AngII.

Overload-induced cardiac hypertrophy was achieved by TAC as previously described (Rockman et al., 1991). Mice were randomly divided into two weight-matched groups: (I) TAC group, n = 6 and (II) sham group, n = 6. Briefly, all of the male mice were anesthetized using gas anesthesia of isoflurane. In the TAC group, the transverse aorta between the right innominate artery and the left carotid artery was narrowed to a 27-gauge needle with 7–0 nylon suture. For the sham group, the same procedure was used on sex- and age-matched mice except that the aorta was not ligated.

After 4 weeks, the mice were anesthetized with pentobarbital sodium and killed. After the heart was perfused with normal saline, the heart was removed, and these tissues were immediately frozen in liquid nitrogen and then stored at −80°C for subsequent studies.

### Quantitative Real-Time PCR

Trizol (Thermo Fisher) was employed to extract total RNA from the tissues. We used a two-step PrimeScript RT reagent Kit (Perfect Real Time, TAKARA) to conduct the reverse transcribing process of mRNA. Based on a miRNA First Strand cDNA Synthesis Tailing Reaction kit (Sangon Biotech), we got the first strand cDNA of miRNA. An Eppendorf Mastercycler^®^ Ep Realplex detection system (Eppendorf, Hamburg, Germany) was used for PCR analysis. Primers were purchased from Sangon Biotech (Shanghai, China) (Supplementary Table S1). The expression levels of mRNAs were normalized to β-actin housekeeping gene. The expression levels of miRNAs were normalized to U6.

### Histopathological Analysis

The heart tissue was fixed in formalin and embedded in 5-μm tissue sections. Sections were subjected to hematoxylin and eosin (H and E) staining for histopathological observation. From the general section, we observe the changes of the heart chamber wall as a whole. The thickness of myocardial fibers was observed in longitudinal and transverse sections under ×400 magnification.

### Statistical Analysis

Data were reported as means ± SD. Student’s *t*-test was applied to test for significant group differences using GraphPad Pro Prism 8 (GraphPad Software, San Diego, CA, United States). The nominal level of *p*-value < 0.05 was chosen as the threshold for determining statistical significance.

## Results

### Identification of Differentially Expressed Genes

**FIGURE 1:**
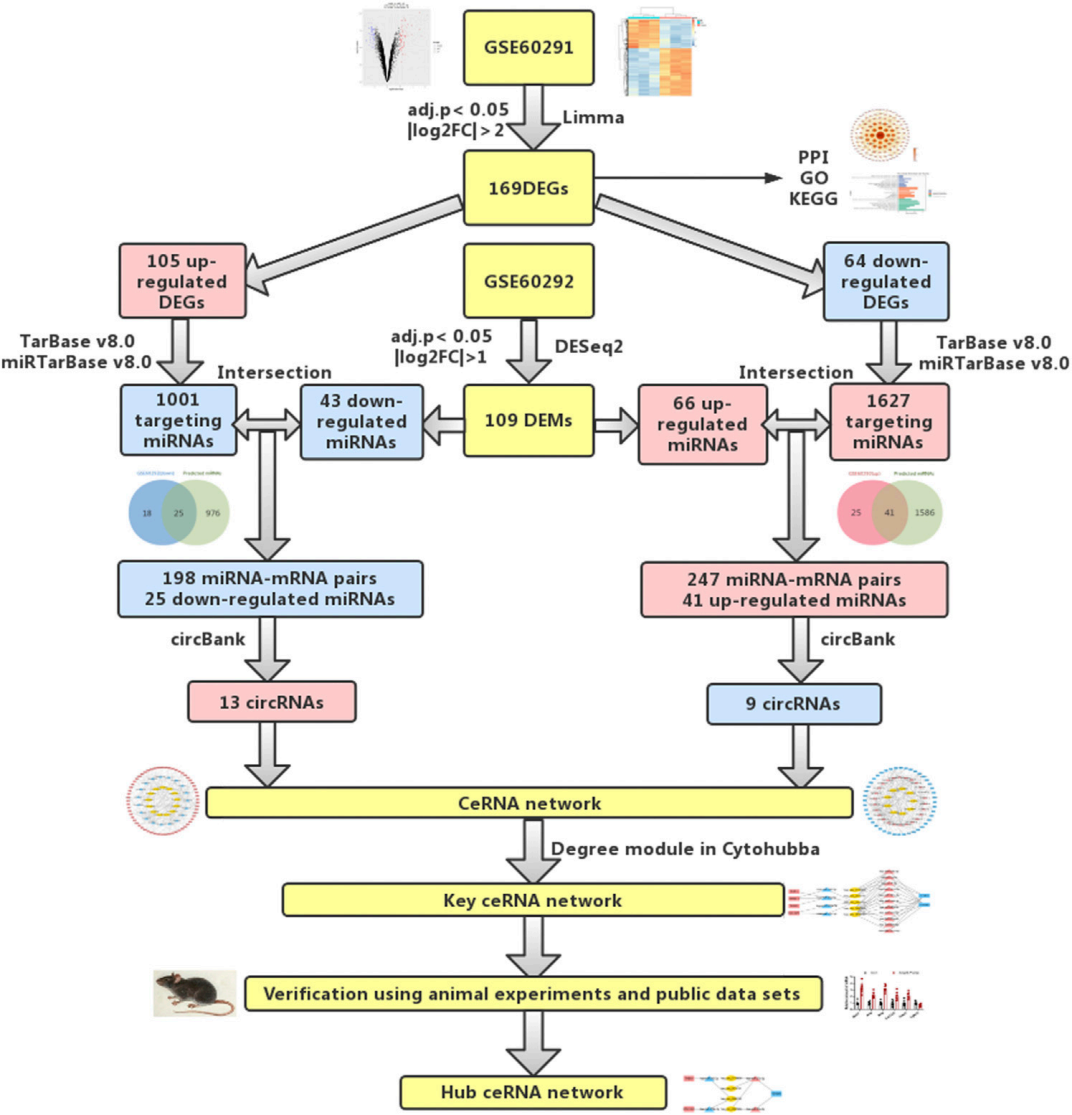
Flowchart of bioinformatics analysis: data collection, processing, analysis, and validation. We screened out differentially expressed mRNAs and miRNAs from public databases. According to online prediction websites, we finally obtained relevant circRNAs and established circRNA-related ceRNA regulatory networks. To verify our results, PCR was used to verify differentially expressed mRNAs and miRNAs in animal cardiac hypertrophy model samples.

**FIGURE 2:**
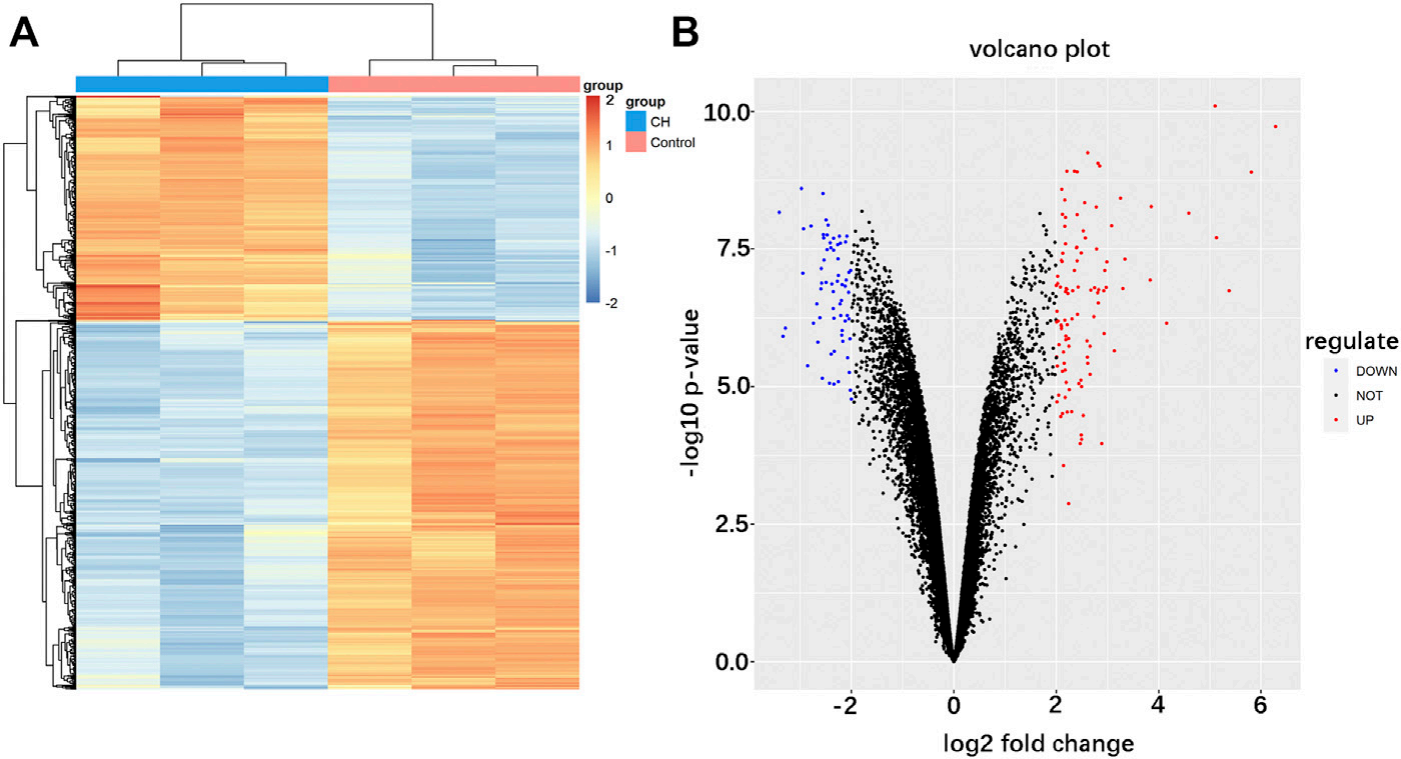
Identification of differentially expressed mRNAs in cardiac hypertrophy. **(A)** Hierarchical clustering heat map of differentially expressed mRNAs. **(B)** Volcano plot of differentially expressed mRNAs. Red represents upregulated mRNAs and green represents downregulated mRNAs.

The analysis procedure of the ceRNA network related to cardiac hypertrophy is described in a flowchart (Figure 1). Using the cut-off criteria of adjusted *p* < 0.05 and |log FC| >2, a total of 172 DEGs, including 106 upregulated genes and 66 downregulated genes, were identified from GSE60291. We deleted three non-coding RNAs (LINC00648, LINC00702, and LOC101928135) after searching the gene type of DEGs from GENE (https://www.ncbi.nlm.nih.gov/gene/). Finally, we obtained 105 upregulated genes and 64 downregulated genes (Supplementary Table S2). All 169 DEGs were displayed with a heat map and a volcano plot (Figures 2A,B). As shown in the figure, the expression level of DEGs well separated the cardiac hypertrophy samples from the control samples.

### Functional Enrichment Analysis of Differentially Expressed Genes

**FIGURE 3:**
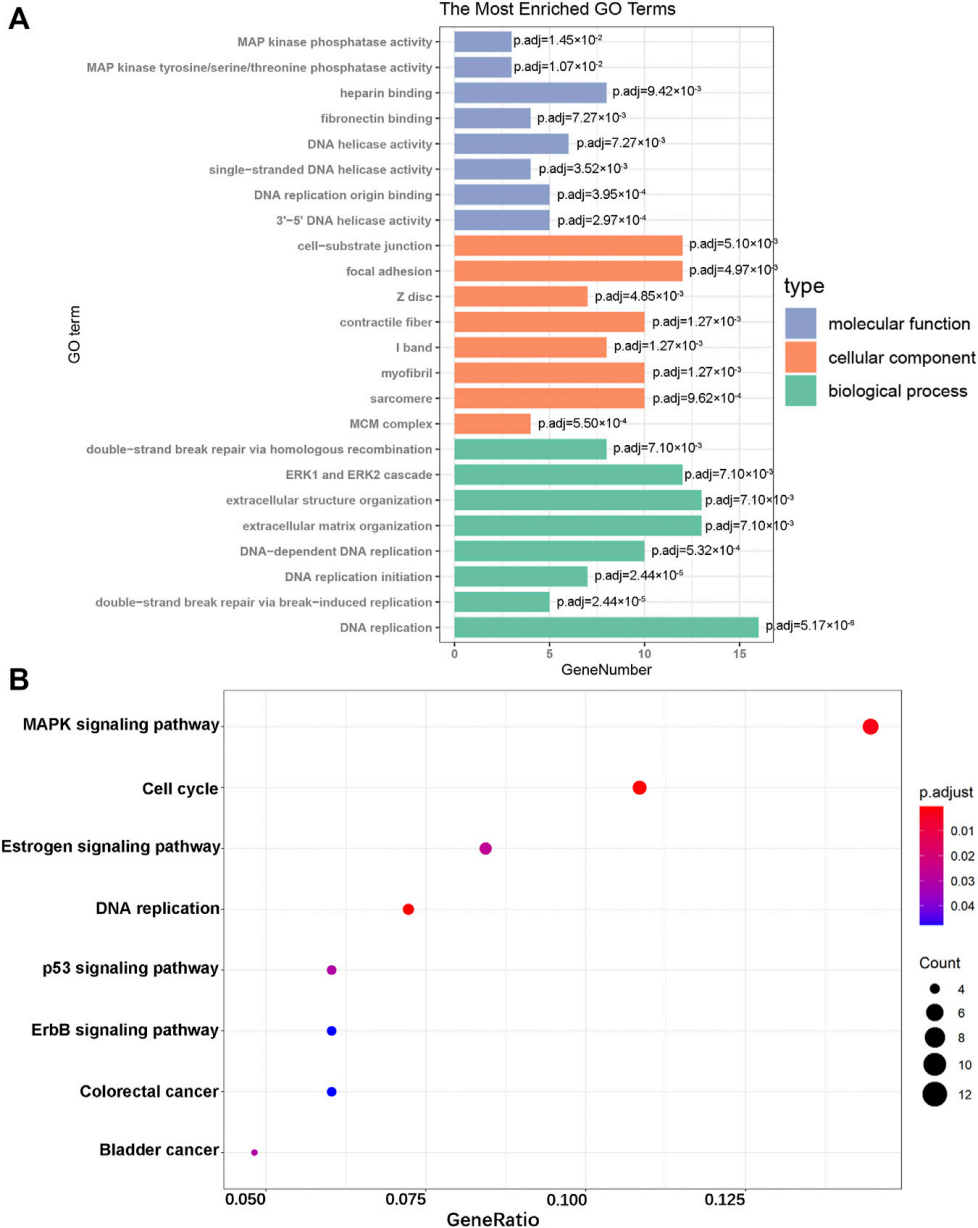
GO enrichment and KEGG pathway analyses. **(A)** GO analysis of the DEGs. **(B)** KEGG pathway analysis of the DEGs.

To further explore the biological functions of DEGs, we divided the results of the GO term into molecular function (MF), cellular component (CC), and biological process (BP). The top eight results in each section are considered the most enriched elements (Figure 3A). The largest number of DEGs enriched in MF was “DNA replication,” those in CC were “cell-substrate junction” and “focal adhesion,” and that in MF was “heparin binding.” Subsequently, KEGG analysis of all DEGs was performed. These genes were mainly enriched in the MAPK signaling pathway, cell cycle, and Estrogen signaling pathway (Figure 3B). As shown, all of these results showed that DEGs were significantly enriched in biological processes related to cardiac hypertrophy.

### Construction of PPI Network

We input all DEGs into the STRING for protein interaction analysis. Connectionless nodes were deleted in the outcome, and we obtained a network with 127 nodes and 444 edges (Supplementary Figure S1). The color and size of the nodes vary with the centrality degree, and the thickness of the edges varies according to the combined score of the edges in Cytoscape software.

### Determination of Differentially Expressed MicroRNAs and Interaction of mRNA-

#### miRNA

**FIGURE 4:**
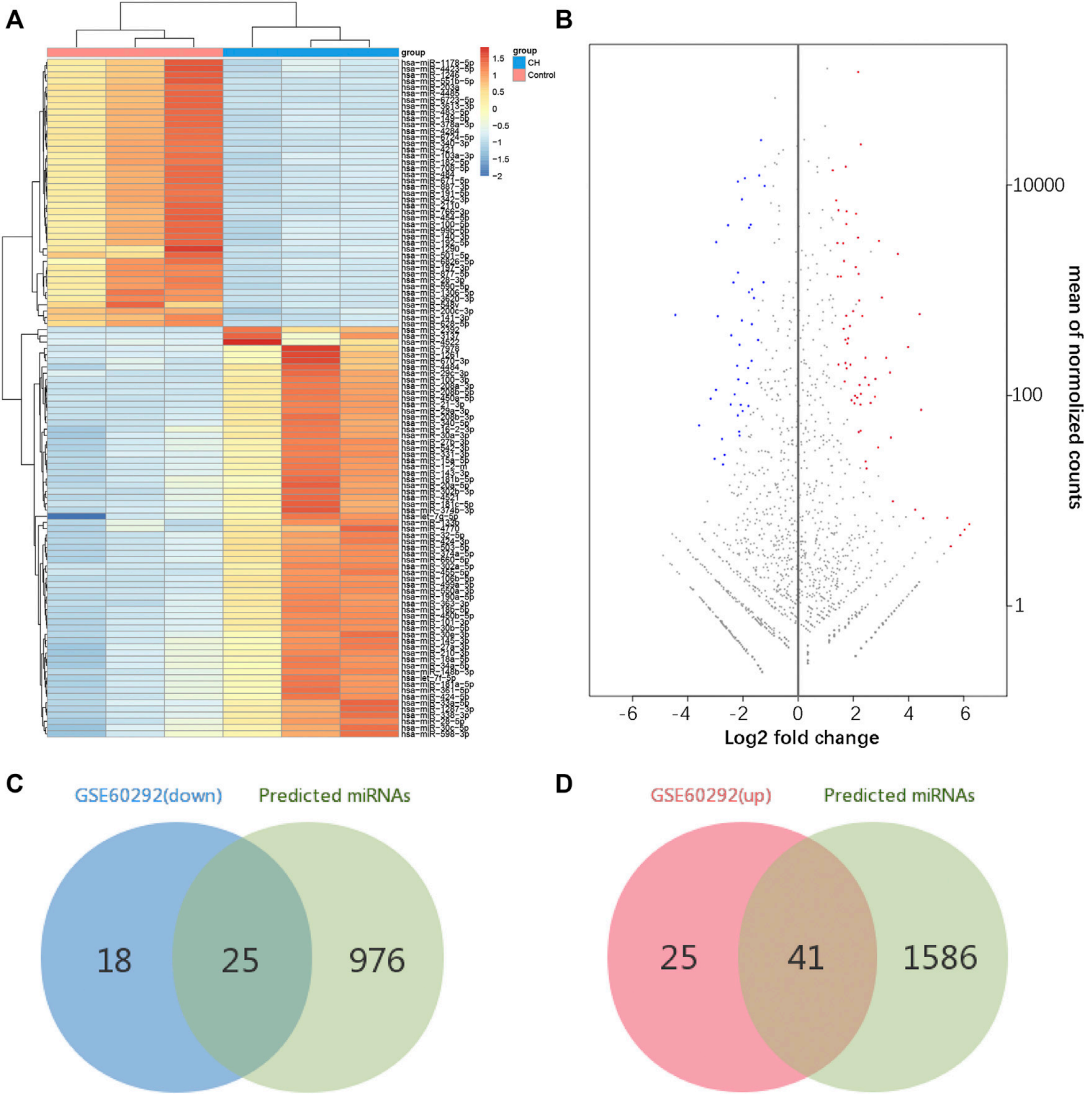
Identification of differentially expressed miRNAs. **(A)** Hierarchical clustering heat map of differentially expressed miRNAs. **(B)** MA plot of differentially expressed miRNAs. **(C,D)** Identification of overlapping miRNAs in DEMs of GSE60292 and predicted miRNAs of DEGs.

According to our screening criteria, we obtained 66 upregulated miRNAs and 43 downregulated miRNAs (Supplementary Table S3). All 109 DEMs were displayed with a heat map and an MA plot (Figures 4A,B). A Venn diagram was used to obtain the intersection of the differentially expressed upregulated miRNAs and the targeting predicted miRNAs of downregulated mRNAs. 41 upregulated miRNAs and 247 miRNA–mRNA pairs were obtained. Through the same procedure, we obtained 25 downregulated miRNAs and 198 miRNA–mRNA pairs (Figures 4C,D).

### Establishment of Key Competing Endogenous RNAs Network

**FIGURE 5:**
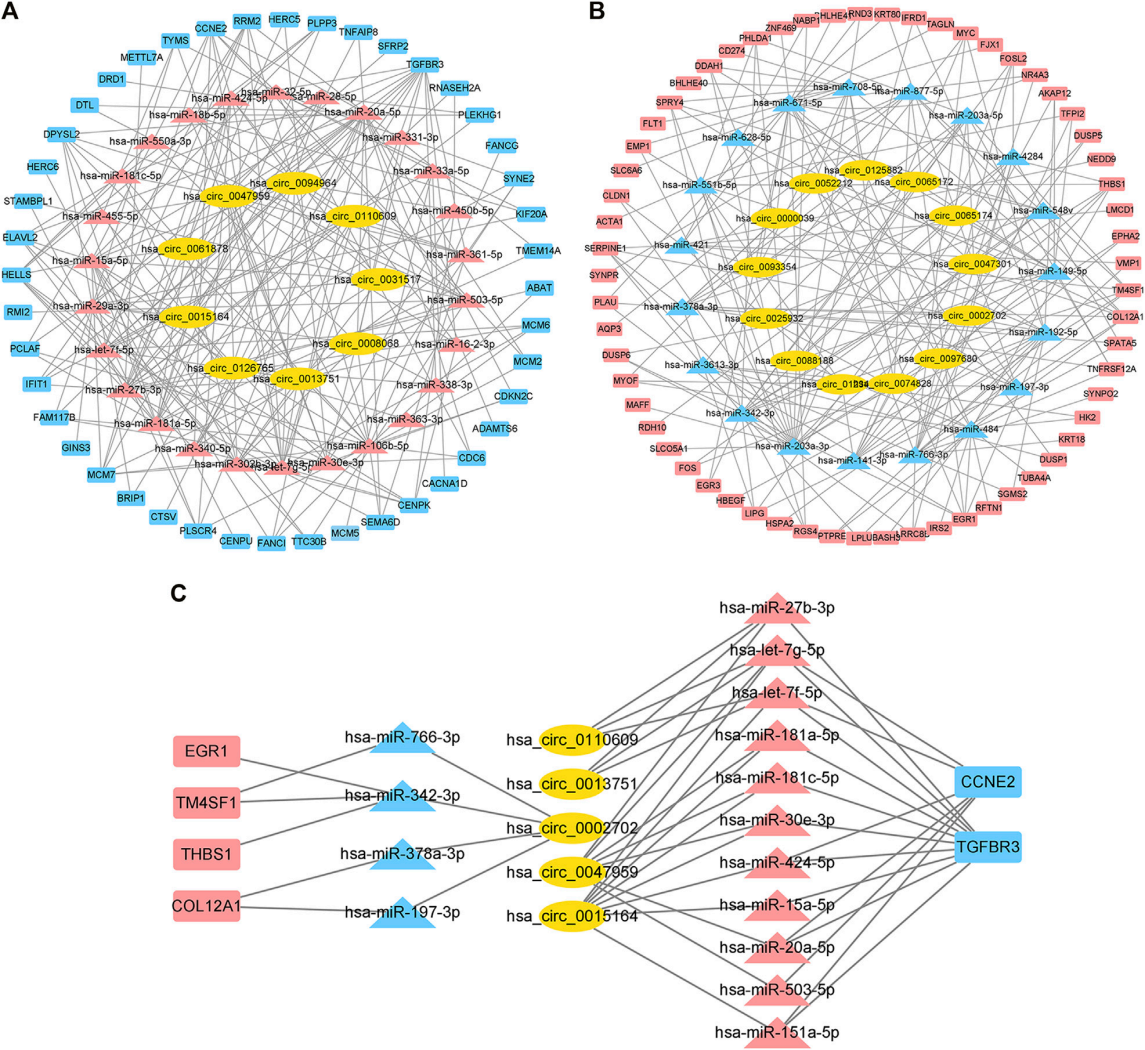
Construction of the ceRNA network and identification of the hub ceRNA network in cardiac hypertrophy. **(A)** Positive regulatory ceRNA network. Yellow represents circRNAs, blue represents downregulated miRNAs, and red represents upregulated mRNAs. **(B)** Negative regulatory ceRNA network. Yellow represents circRNAs, red represents upregulated miRNAs, and blue represents downregulated mRNAs. **(C)** Key ceRNA network in cardiac hypertrophy.

We used CircBank to predict targeting circRNAs. By ranking the circRNAs according to the predicted times, we ultimately obtained 22 circRNAs associated with cardiac hypertrophy (Supplementary Table S4). Among them, 13 circRNAs were in the positively regulated ceRNA network and 9 circRNAs were in the negatively regulated ceRNA network. These circRNA–miRNA pairs were linked to the previous miRNA–mRNA pairs, and the unconnected parts were removed, resulting in two independent circRNA-miRNA-mRNA ceRNA regulatory networks (Figures 5A,B). Using the aforementioned method, we ultimately obtained a positive regulatory key network (4 mRNAs, 4 miRNAs, and 1 circRNA) and a negative regulatory key network (2 mRNAs, 11 miRNAs, and 4 circRNAs) (Figure 5C).

### Experimental Verification of Hub Competing Endogenous RNAs Network

**FIGURE 6:**
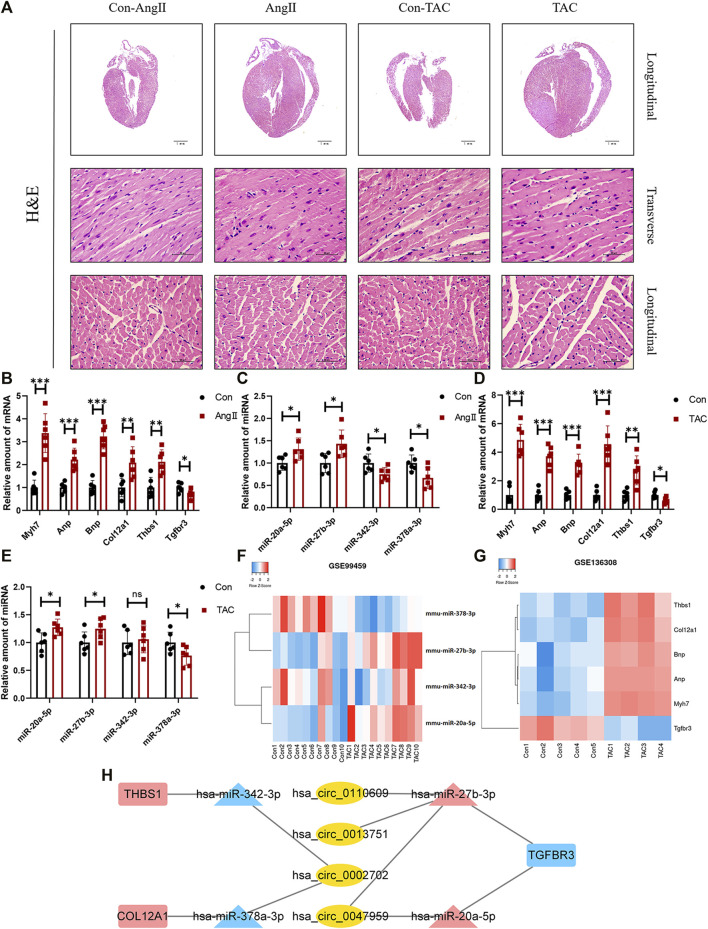
Confirmation of microarray results in animal models of myocardial hypertrophy. **(A)** Representative images of H&E staining (400×). **(B–E)** Expression of Myh7, Anp, Bnp, Col12a1, Thbs1, Tgfbr3, miR-20a-5p, miR-27b-3p, miR-342-3p, and miR-378a-3p in animal models of cardiac hypertrophy. (n = 6 per group, *p-value <0.05, ** *p*-value <0.01, ***p-value <0.001 versus AngII group or TAC group.) **(F,G)** Expression of Col12a1, Thbs1, Tgfbr3, miR-20a-5p, miR-27b-3p, miR-342-3p, and miR-378a-3p in GSE99459 and GSE136308. **(H)** Hub ceRNA network.

HE staining revealed that compared with the corresponding control group, the heart of the AngII group and the transverse aortic constriction (TAC) group was significantly larger than that of the corresponding control group. The myocardial fibers were obviously thickened (Figure 6A). Both AngII group and TAC group showed that the expression levels of Myh7, Anp, and Bnp, which can reflect the degree of myocardial hypertrophy, were significantly increased. At the same time, the expression levels of three mRNAs (Col12a1, Thbs1, and Tgfbr3) and four miRNAs (miR-20a-5p, miR-27b-3p, miR-342-3p, and miR-378a-3p) were significantly different (Figures 6B,C). These trends were consistent with our analysis.

Except for miR-342-3p, the expression trends of these mRNAs and miRNAs in the TAC group were the same as those for the AngII group (Figures 6D,E). These results were further confirmed by GSE99459 and GSE136308 (Figures 6F,G). We finally obtained a hub ceRNA network which was verified by animal experiments (Figure 6H) and differentially expressed.

## Discussion

In our study, we screened out DEGs and DEMs based on data from the public datasets GSE60291 and GSE60292, respectively. We set up a PPI network for the obtained DEGs. It is worth noting that several known proteins associated with myocardial hypertrophy, such as Myc, Fos, and Egr1, were present at the center of the network (Brand et al., 1993; Ahuja et al., 2010; Palomer et al., 2020). In addition, other proteins in the network have also been shown to be implicated in cardiovascular diseases. Scott D Shapiro et al. found that exogenous injection of CCNA2 virus can induce myocardial regeneration after infarction through cytoplasmic division of adult cardiomyocytes (Shapiro et al., 2014). Jin et al. found that DUSP1 reduces cardiac ischemia/reperfusion injury by inhibiting Mff-mediated mitochondrial fission and BNIP3-related mitochondrial autophagy through the JNK pathway (Jin et al., 2018). To a certain extent, these research results indicated that the DEGs screened by us can well reflect the differentially expressed proteins in cardiac hypertrophy. This point was further confirmed in the results of GO analysis and KEGG analysis. These results showed that DEGs were concentrated in several classical pathways, including the MAPK pathway, the estrogen receptor pathway, and the p53 signaling pathway. In addition, the erbB signaling pathway also appeared in the results. ErbB signaling has been associated with cancer in previous studies (Yarden and Sliwkowski, 2001). In recent years, more and more studies have shown that the erbB signaling pathway plays an important role in cardiac hypertrophy and heart failure. Blockage of erbB-1 signaling in the heart leads to blockage of erbB-2 signaling, and blockage of both leads to cardiac dysfunction (Rajagopalan et al., 2008). In neonatal, adolescent, and adult cardiomyocytes, cardiac hypertrophy is caused by induction of constitutively active ERBB2 (D'Uva et al., 2015). Therefore, based on our results, we hypothesize that the erbB-related signaling pathway will become a potential research direction of cardiac hypertrophy in the future.

The ceRNA network has been described as a complex post-transcriptional endogenous regulatory network in which circRNAs, lncRNAs, and other RNAs act as sponges for miRNAs to regulate mRNA expression. LncRNA ZFAS1 promotes the transformation of lung fibroblasts into myofibroblasts and iron function through the miR-150-5p/SLC38A1 axis (Yang et al., 2020). LncRNA Hoxaas3 regulates Runx1 by targeting miR-450b-5p and promotes lung fibroblast activation and fibrosis (Lin et al., 2020). In the ceRNA network, circRNA, as the only circular non-coding RNA, can compete with the targeting mRNA of related miRNA through its own miRNAs elements, ultimately affecting the mRNA expression level (Arnaiz et al., 2019). We used the public website prediction tool to predict the targets of DEGs and DEMs, and through the aforementioned screening method, we ultimately obtained a positive regulatory ceRNA network and a negative regulatory ceRNA network. Then, the Cytohubba plug-in of Cytoscape was used to further screen the key ceRNA network. The expression levels of mRNAs and miRNAs in the network were verified by animal experiments. Finally, three differentially expressed mRNAs (Col12a1, Thbs1, and Tgfbr3) and four differentially expressed miRNAs (miR-20a-5p, miR-27b-3p, miR-342-3p, and miR-378a-3p) were obtained.

In the heart, Col12a1 usually appears as a marker of myocardial fibrosis (Ning et al., 2017). Our results suggested that Col12a1 may play a key part in the process of cardiac hypertrophy through a ceRNA regulatory network. In addition, Thbs1 is a physiological regulator of TGFβ activation and is involved in the regulation of the myocardial fibrosis–related ceRNA network. One study shows that LncRNA RNF7 can promote cardiac fibrosis *via* the miR-543/Thbs1 axis and activation of TGFβ1 (Ouyang et al., 2020). In some AngII-induced cardiovascular disease models, the expression level of THBS1 was significantly increased (Bao et al., 2020; Jana et al., 2020). This trend is consistent with our results and further supports that Thbs1 may be a key regulatory site in the cardiac hypertrophy–related ceRNA network. In contrast to Thbs1, Tgfbr3 is a potential negative regulator of the TGFβ signaling pathway. The expression level of Tgfbr3 is negatively correlated with the degree of myocardial fibrosis (Chu et al., 2011). Overexpression of Tgfbr3 reduces collagen production in fibroblasts by inhibiting miR-21 expression (Liang et al., 2012). In addition, Tgfbr3 is an important drug target. A drug study has shown that Simvastatin could reduce cardiac fibrosis induced by infarction *via* upregulating the expression of Tgfbr3 (Sun et al., 2015).

Relevant miRNAs were also acquired. A study also indicated that miR-27b-3p was significantly increased in auricular tissue and angII-stimulated atrial fibroblasts in patients with atrial fibrillation. In this study, it is worth noting that miR-27b-3p promotes atrial fibrosis by targeting Tgfbr3 to activate Smad3 signaling in atrial fibroblasts (Yang et al., 2019). The targeting interaction relationship between miR-27b-3p and Tgfbr3 appeared in our prediction results, which further indicated that the miR-27b-3p/Tgfbr3 axis may play a significant role in myocardial hypertrophy. The expression level of plasma miR-20a-5p was proportional to the degree of left ventricular remodeling and dilation after myocardial infarction (Gao et al., 2019). The level of miR-342-3p in circulating miRNAs in heart failure model mice was significantly reduced (Kaneko et al., 2017). MiR-378a-3p could prevent myocardial apoptosis induced by ischemia-reperfusion injury through TRIM55/DUSP1/JNK signaling (Tan et al., 2020). These proven miRNAs might provide therapeutic targets for myocardial hypertrophy.

In recent years, circRNA has attracted increased scholarly attention in the study of myocardial hypertrophy. Garikipati et al. showed that the circRNA circFNDC3b regulates cardiac repair after myocardial infarction *via* the FUS/VEGF-A axis (Garikipati et al., 2019). Gan et al. confirmed that the circRNA_101,237 mediates anoxia/reoxygenation injury by targeting let-7a-5p/IGF2BP3 in cardiomyocytes (Gan et al., 2020). The samples in GSE60291 and GSE60292 belong to *Homo sapiens*. The species specificity of circRNAs may account for the lack of suitable experimental methods to prove the expression level of circRNAs selected in animal experiments. But the expression levels of these circRNA-related mRNAs and miRNAs in our ceRNA hub network have been proved by PCR. Therefore, the circRNAs (circ_0002702, circ_0110,609, circ_0013751, and circ_0047959) predicted on this basis may play a key role in the process of myocardial hypertrophy, providing a new inspiration for the clinical diagnosis and treatment of myocardial hypertrophy.

In conclusion, our study established and analyzed the circRNA-related ceRNA network involved in cardiac hypertrophy disorders for the first time and successively verified the differentially expressed miRNAs and mRNAs in animal experiments. The analysis results provided a new therapeutic target and a new idea for the regulatory mechanism of the cardiac hypertrophy–related ceRNA network. Nonetheless, to further clarify the expression levels of these RNAs in cardiac hypertrophy, it is still necessary to verify the ceRNAs with clinical samples and reveal the specific regulatory mechanism of the ceRNA network related to cardiac hypertrophy.

## Data Availability

The datasets presented in this study can be found in online repositories. The names of the repository/repositories and accession number(s) can be found below: https://www.ncbi.nlm.nih.gov/, GSE60291 https://www.ncbi.nlm.nih.gov/, GSE60292.
