# Supplementary material for: Integrated Analysis of circRNA-miRNA-mRNA ceRNA Network in Cardiac Hypertrophy
Source: Front Genet. 2022 Feb 8;13:781676. doi: 10.3389/fgene.2022.781676 (PMC8860901; doi:10.3389/fgene.2022.781676)
Supplement: Supplementary file 1 [file Table1.DOCX]

**Table S1: qPCR primer sequences.**

| *Gene Name* | *Primers (5` - 3`)* |
| --- | --- |
| *β-acting* | F: CTACCTCATGAAGATCCTGACC  R: CACAGCTTCTCTTTGATGTCAC |
| *Myhc* | F: CAGAACACCAGCCTCATCAACCAG  R: TTCTCCTCTGCGTTCCTACACTCC |
| *Anp* | F: AAGAACCTGCTAGACCACCTGGAG  R: TGCTTCCTCAGTCTGCTCACTCAG |
| *Bnp* | F: TGCTGGAGCTGATAAGAGAAAA  R: GAAGGACTCTTTTTGGGTGTTC |
| *Col12a1* | F: ACCAACAGAGGCTCCTACAGAACC  R: CTCCAAGAGGCATCCGTCAAGAAC |
| *Thbs1* | F: ATGCCTGCGATGATGACGATGAC  R: CTGGGCTGGGTTGTAATGGAATGG |
| *Tgfbr3* | F: AAGTGGACAAGAAGCGGTTCAGC  R: AGGCGTCGTCAGGAGTCACAC |
| *Mmu-miR-20a-5p* | CGCGTAAAGTGCTTATAGTGCAGGTAG |
| *Mmu-miR-27b-3p* | CCGTTCACAGTGGCTAAGTTCTGC |
| *Mmu-miR378a-3p* | CGACTGGACTTGGAGTCAGAAGG |
| *Mmu-miR-342-3p* | TATCTCACACAGAAATCGCACCCGT |
