# Supplementary material for: Integrated Analysis of circRNA-miRNA-mRNA ceRNA Network in Cardiac Hypertrophy
Source: Front Genet. 2022 Feb 8;13:781676. doi: 10.3389/fgene.2022.781676 (PMC8860901; doi:10.3389/fgene.2022.781676)
Supplement: Supplementary file 2 [file Table3.DOC]

**Table S3: Differentially expressed miRNAs.**

| *name* | *log2FoldChange* | *padj* |
| --- | --- | --- |
| *hsa-miR-2392* | 6.201800242 | 0.002684275 |
| *hsa-miR-3137* | 6.029439707 | 0.008253393 |
| *hsa-miR-100-3p* | 5.874842213 | 0.008119851 |
| *hsa-miR-1261* | 5.530702149 | 0.030321914 |
| *hsa-miR-4522* | 5.405686566 | 0.021856648 |
| *hsa-miR-670-3p* | 4.531243794 | 0.042742944 |
| *hsa-miR-450a-5p* | 4.45807886 | 7.77E-17 |
| *hsa-miR-499a-5p* | 4.403634486 | 4.89E-85 |
| *hsa-miR-7978* | 4.241173188 | 0.021856648 |
| *hsa-miR-21-3p* | 3.984137493 | 3.91E-52 |
| *hsa-miR-208a-3p* | 3.613593976 | 2.76E-108 |
| *hsa-miR-4770* | 3.432165358 | 0.042742944 |
| *hsa-miR-181c-5p* | 3.361408492 | 9.13E-07 |
| *hsa-miR-208b-5p* | 3.328847979 | 4.14E-24 |
| *hsa-miR-340-5p* | 3.191676703 | 6.62E-26 |
| *hsa-miR-208b-3p* | 3.034099317 | 1.78E-46 |
| *hsa-miR-106b-5p* | 2.929222043 | 2.79E-76 |
| *hsa-miR-424-3p* | 2.898410233 | 0.00011519 |
| *hsa-miR-550a-3p* | 2.800583593 | 3.80E-16 |
| *hsa-miR-374a-5p* | 2.782981283 | 5.66E-11 |
| *hsa-miR-450b-5p* | 2.625663351 | 1.38E-08 |
| *hsa-miR-29a-3p* | 2.560849014 | 5.75E-11 |
| *hsa-miR-374b-3p* | 2.479390896 | 0.035308396 |
| *hsa-miR-1287-3p* | 2.447447663 | 0.01820666 |
| *hsa-miR-363-3p* | 2.441146983 | 4.14E-14 |
| *hsa-miR-338-3p* | 2.429491191 | 2.95E-10 |
| *hsa-miR-18b-5p* | 2.326273148 | 1.19E-24 |
| *hsa-miR-190a-5p* | 2.272153596 | 3.78E-07 |
| *hsa-miR-143-3p* | 2.271195054 | 8.07E-31 |
| *hsa-miR-29c-3p* | 2.264372576 | 0.008349516 |
| *hsa-miR-4521* | 2.259146263 | 5.28E-05 |
| *hsa-miR-33a-5p* | 2.224918033 | 5.77E-07 |
| *hsa-miR-503-5p* | 2.213597621 | 9.46E-32 |
| *hsa-miR-455-5p* | 2.196136259 | 0.00514966 |
| *hsa-miR-15a-5p* | 2.193624376 | 1.19E-28 |
| *hsa-miR-1-2-m* | 2.174966997 | 6.20E-31 |
| *hsa-miR-101-3p* | 2.170274855 | 3.06E-50 |
| *hsa-miR-4484* | 2.139293354 | 9.53E-05 |
| *hsa-miR-30b-5p* | 2.100205061 | 1.27E-52 |
| *hsa-miR-660-5p* | 2.08791419 | 4.19E-35 |
| *hsa-miR-16-2-3p* | 2.060470323 | 0.000557029 |
| *hsa-miR-302a-5p* | 2.040719214 | 0.000593851 |
| *hsa-miR-148b-3p* | 1.993036152 | 1.47E-10 |
| *hsa-miR-133b* | 1.933848671 | 0.004277314 |
| *hsa-miR-32-5p* | 1.895227287 | 1.07E-06 |
| *hsa-miR-302b-3p* | 1.87654891 | 9.68E-07 |
| *hsa-miR-542-3p* | 1.813483554 | 3.37E-08 |
| *hsa-miR-30e-3p* | 1.784678148 | 3.01E-06 |
| *hsa-miR-30a-3p* | 1.757094819 | 9.97E-05 |
| *hsa-let-7f-5p* | 1.755403418 | 2.26E-13 |
| *hsa-let-7g-5p* | 1.745989793 | 6.32E-15 |
| *hsa-miR-361-5p* | 1.742621982 | 1.03E-06 |
| *hsa-miR-27b-3p* | 1.725926263 | 1.12E-18 |
| *hsa-miR-28-5p* | 1.714965469 | 4.38E-06 |
| *hsa-miR-331-3p* | 1.714418915 | 0.000309447 |
| *hsa-miR-145-3p* | 1.685809874 | 0.009320139 |
| *hsa-miR-20a-5p* | 1.661203244 | 2.15E-05 |
| *hsa-miR-424-5p* | 1.648831543 | 4.77E-05 |
| *hsa-miR-181a-5p* | 1.632793057 | 2.85E-09 |
| *hsa-miR-210-3p* | 1.547728487 | 9.57E-08 |
| *hsa-miR-598-3p* | 1.460581037 | 0.049225562 |
| *hsa-miR-18a-5p* | 1.459569001 | 9.12E-08 |
| *hsa-miR-181b-5p* | 1.438828988 | 0.001207182 |
| *hsa-miR-27a-3p* | 1.423147285 | 8.38E-06 |
| *hsa-miR-34a-5p* | 1.380291033 | 2.94E-05 |
| *hsa-miR-30c-5p* | 1.253185951 | 0.002010081 |
| *hsa-miR-99b-5p* | -1.21386272 | 0.007846729 |
| *hsa-miR-140-3p* | -1.253334918 | 0.031851188 |
| *hsa-miR-103a-3p* | -1.343980817 | 1.20E-05 |
| *hsa-miR-100-5p* | -1.41112579 | 3.31E-09 |
| *hsa-miR-1306-5p* | -1.450999633 | 0.042742944 |
| *hsa-miR-590-5p* | -1.600521199 | 7.07E-08 |
| *hsa-miR-197-3p* | -1.668699789 | 8.52E-11 |
| *hsa-miR-192-5p* | -1.678029094 | 3.68E-07 |
| *hsa-miR-182-5p* | -1.68631632 | 0.000316478 |
| *hsa-miR-484* | -1.71784652 | 4.25E-17 |
| *hsa-miR-149-5p* | -1.775700148 | 2.19E-18 |
| *hsa-miR-28-3p* | -1.780535453 | 5.24E-15 |
| *hsa-miR-454-5p* | -1.80134484 | 0.013550621 |
| *hsa-miR-766-3p* | -1.811456368 | 0.000126319 |
| *hsa-miR-501-5p* | -1.850564987 | 0.004337552 |
| *hsa-miR-191-5p* | -1.929691147 | 2.98E-41 |
| *hsa-miR-877-5p* | -2.009939816 | 0.002010081 |
| *hsa-miR-342-3p* | -2.034483456 | 1.47E-49 |
| *hsa-miR-340-3p* | -2.036874358 | 5.71E-16 |
| *hsa-miR-2110* | -2.080440419 | 0.00049709 |
| *hsa-miR-4423-5p* | -2.12283997 | 0.021082799 |
| *hsa-miR-628-5p* | -2.122931202 | 1.73E-10 |
| *hsa-miR-483-5p* | -2.137564629 | 0.008224849 |
| *hsa-miR-1178-5p* | -2.169083795 | 9.14E-05 |
| *hsa-miR-887-3p* | -2.178861777 | 1.61E-31 |
| *hsa-miR-378a-3p* | -2.18356118 | 3.76E-43 |
| *hsa-miR-6723-5p* | -2.186317663 | 0.000625486 |
| *hsa-miR-671-5p* | -2.208322845 | 1.85E-09 |
| *hsa-miR-3620-3p* | -2.300660987 | 1.22E-06 |
| *hsa-miR-1246* | -2.344653807 | 7.73E-27 |
| *hsa-miR-708-5p* | -2.42343099 | 3.40E-20 |
| *hsa-miR-1290* | -2.443235009 | 3.13E-05 |
| *hsa-miR-4284* | -2.539437892 | 1.62E-87 |
| *hsa-miR-200c-3p* | -2.663149126 | 0.013550621 |
| *hsa-miR-6826-5p* | -2.721892368 | 0.012086467 |
| *hsa-miR-6724-5p* | -2.757137528 | 0.000126319 |
| *hsa-miR-4485* | -2.91292841 | 1.65E-45 |
| *hsa-miR-421* | -2.969263376 | 2.92E-116 |
| *hsa-miR-3613-3p* | -2.976290014 | 5.46E-13 |
| *hsa-miR-548v* | -3.027187603 | 0.002010081 |
| *hsa-miR-551b-5p* | -3.1708992 | 1.98E-12 |
| *hsa-miR-141-3p* | -3.587981788 | 4.46E-08 |
| *hsa-miR-203a* | -4.448157312 | 9.86E-92 |
