# Supplementary figures and images for: Integrated Analysis of circRNA-miRNA-mRNA ceRNA Network in Cardiac Hypertrophy
Source: Front Genet. 2022 Feb 8;13:781676. doi: 10.3389/fgene.2022.781676 (PMC8860901; doi:10.3389/fgene.2022.781676)

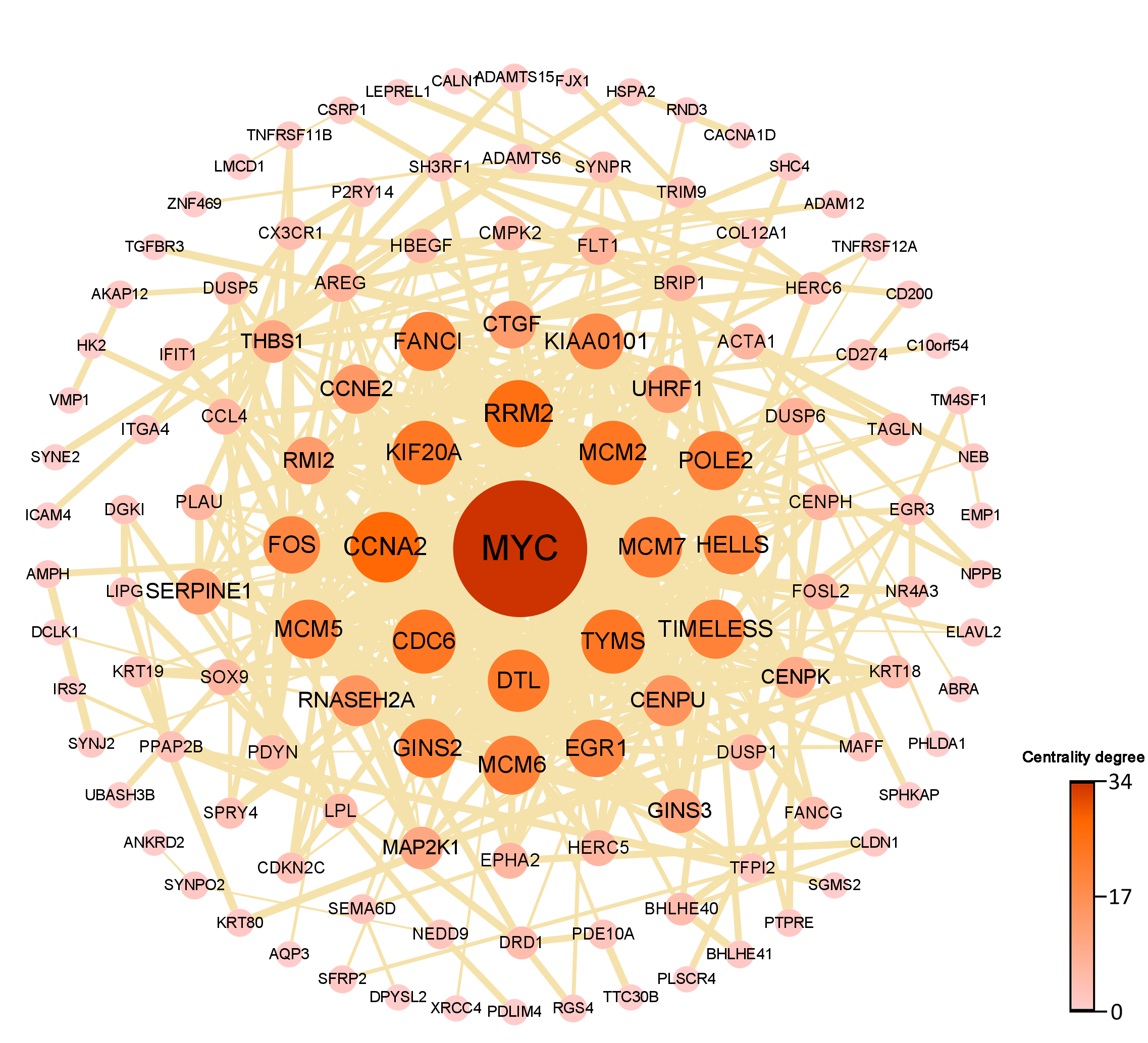

Supplement: Supplementary file 3 [file Image1.TIF]
