# Supplementary material for: Integrated Analysis of circRNA-miRNA-mRNA ceRNA Network in Cardiac Hypertrophy
Source: Front Genet. 2022 Feb 8;13:781676. doi: 10.3389/fgene.2022.781676 (PMC8860901; doi:10.3389/fgene.2022.781676)
Supplement: Supplementary file 4 [file Table2.DOCX]

**Table S2: Differentially expressed mRNAs.**

| *gene name* | *logFC* | *adj.P.Val* | *change* |
| --- | --- | --- | --- |
| *C4orf54* | 6.28270813 | 1.88E-06 | UP |
| *CCL4* | 5.813088689 | 2.84E-06 | UP |
| *MAFF* | 5.3743312 | 1.91E-05 | UP |
| *RGS4* | 5.13264987 | 8.72E-06 | UP |
| *SERPINE1* | 5.103879496 | 1.59E-06 | UP |
| *ATP6V1FNB* | 4.59002575 | 6.59E-06 | UP |
| *DCLK1* | 4.156506379 | 3.87E-05 | UP |
| *SOX9* | 3.855254666 | 6.42E-06 | UP |
| *ANKRD2* | 3.838616741 | 1.64E-05 | UP |
| *CCN2* | 3.344817825 | 1.18E-05 | UP |
| *HK2* | 3.299552717 | 1.85E-05 | UP |
| *NAV3* | 3.252704136 | 5.85E-06 | UP |
| *ABRA* | 3.136369944 | 7.20E-05 | UP |
| *FOSL2* | 3.084938008 | 8.03E-06 | UP |
| *SH3RF1* | 2.991853595 | 1.20E-05 | UP |
| *PHLDA1* | 2.972193307 | 1.78E-05 | UP |
| *IL17B* | 2.949167147 | 1.36E-05 | UP |
| *CCN1* | 2.934362184 | 4.90E-05 | UP |
| *DUSP6* | 2.929280263 | 1.91E-05 | UP |
| *VMP1* | 2.885706152 | 0.000933435 | UP |
| *MAP2K1* | 2.8493298 | 2.84E-06 | UP |
| *ITGA4* | 2.827442574 | 1.90E-05 | UP |
| *PCDH8* | 2.824899127 | 2.52E-05 | UP |
| *ENPEP* | 2.811898889 | 2.84E-06 | UP |
| *DGKI* | 2.805712668 | 1.99E-05 | UP |
| *THBS1* | 2.791306097 | 9.83E-06 | UP |
| *FLT1* | 2.78101455 | 6.42E-06 | UP |
| *PTPRE* | 2.750716463 | 1.80E-05 | UP |
| *LIPG* | 2.735730838 | 3.35E-05 | UP |
| *XRCC4* | 2.674575871 | 1.91E-05 | UP |
| *DUSP1* | 2.664459383 | 6.55E-05 | UP |
| *TAGLN* | 2.662553921 | 0.000129232 | UP |
| *TRIM9* | 2.612652876 | 9.87E-05 | UP |
| *RDH10* | 2.61264317 | 2.84E-06 | UP |
| *TNFRSF11B* | 2.608974774 | 8.50E-05 | UP |
| *SHC4* | 2.601854457 | 5.69E-05 | UP |
| *IFRD1* | 2.565840943 | 8.72E-06 | UP |
| *LRRC8B* | 2.553445539 | 6.13E-06 | UP |
| *LPL* | 2.530017724 | 8.45E-06 | UP |
| *NPPB* | 2.495144093 | 0.000814781 | UP |
| *EGR3* | 2.494404587 | 0.000178047 | UP |
| *AREG* | 2.490950958 | 0.000713579 | UP |
| *CLDN1* | 2.490406321 | 1.03E-05 | UP |
| *SLCO5A1* | 2.481846411 | 0.000149666 | UP |
| *ACTA1* | 2.469191937 | 0.000930855 | UP |
| *KRT19* | 2.443407182 | 0.000164933 | UP |
| *SGMS2* | 2.427000374 | 1.78E-05 | UP |
| *RND3* | 2.414076925 | 3.19E-05 | UP |
| *NR4A3* | 2.410926813 | 1.18E-05 | UP |
| *CD200* | 2.406584316 | 6.59E-06 | UP |
| *PDLIM4* | 2.405134137 | 2.84E-06 | UP |
| *LMCD1* | 2.402358044 | 9.54E-06 | UP |
| *DUSP5* | 2.396849034 | 9.54E-06 | UP |
| *IRS2* | 2.357617544 | 2.84E-06 | UP |
| *CSRP1* | 2.355325876 | 1.36E-05 | UP |
| *SYNJ2* | 2.321212425 | 1.91E-05 | UP |
| *RFTN1* | 2.305409125 | 3.52E-05 | UP |
| *KRT80* | 2.301721916 | 0.000356025 | UP |
| *EMP1* | 2.256431948 | 0.000193536 | UP |
| *P3H2* | 2.248401997 | 6.55E-05 | UP |
| *TM4SF1* | 2.241780138 | 0.006332824 | UP |
| *UBASH3B* | 2.238346373 | 5.47E-05 | UP |
| *AQP3* | 2.230142142 | 2.01E-05 | UP |
| *MYC* | 2.217306483 | 0.000360014 | UP |
| *NIPAL4* | 2.212205649 | 4.03E-05 | UP |
| *ZNF469* | 2.20989681 | 1.89E-05 | UP |
| *FJX1* | 2.206770631 | 2.84E-06 | UP |
| *KRT18* | 2.197180583 | 1.85E-05 | UP |
| *NEB* | 2.195164751 | 0.00015996 | UP |
| *LOXL4* | 2.188757821 | 5.19E-05 | UP |
| *HBEGF* | 2.186386977 | 5.58E-05 | UP |
| *TRIM58* | 2.180036265 | 1.97E-05 | UP |
| *AKAP12* | 2.179121615 | 8.99E-06 | UP |
| *CD274* | 2.178520647 | 7.10E-06 | UP |
| *MICALCL* | 2.174199915 | 0.000116545 | UP |
| *BHLHE41* | 2.172747274 | 0.000242586 | UP |
| *FOS* | 2.170585279 | 3.63E-05 | UP |
| *PDYN* | 2.167775014 | 8.03E-06 | UP |
| *SPRY4* | 2.16615116 | 5.90E-06 | UP |
| *BHLHE40* | 2.151754263 | 9.87E-05 | UP |
| *SLC6A6* | 2.143161498 | 0.001862531 | UP |
| *GADD45G* | 2.139043478 | 7.44E-05 | UP |
| *VSIR* | 2.129278633 | 1.03E-05 | UP |
| *NANOS1* | 2.123202686 | 6.59E-06 | UP |
| *SPATA5* | 2.122712068 | 0.000119985 | UP |
| *ADAM12* | 2.120757478 | 0.000371998 | UP |
| *COL12A1* | 2.114445203 | 1.18E-05 | UP |
| *SYNPO2* | 2.109538115 | 4.77E-06 | UP |
| *NEDD9* | 2.104175541 | 1.18E-05 | UP |
| *NABP1* | 2.10101313 | 4.24E-05 | UP |
| *MYOF* | 2.099490102 | 4.44E-05 | UP |
| *TNFRSF12A* | 2.096720333 | 4.05E-05 | UP |
| *EGLN3* | 2.087789247 | 0.000409824 | UP |
| *PLAU* | 2.082205862 | 1.78E-05 | UP |
| *C11orf96* | 2.054019088 | 6.20E-05 | UP |
| *EGR1* | 2.045720583 | 0.000226524 | UP |
| *TFPI2* | 2.035665952 | 3.68E-05 | UP |
| *DDAH1* | 2.030485748 | 1.56E-05 | UP |
| *EPHA2* | 2.025992721 | 1.71E-05 | UP |
| *PIK3AP1* | 2.023623836 | 0.000105713 | UP |
| *SYNPR* | 2.01701125 | 0.00026968 | UP |
| *TUBA4A* | 2.012911374 | 2.98E-05 | UP |
| *ADAMTS15* | 2.011325283 | 3.81E-05 | UP |
| *PDE10A* | 2.005435286 | 8.47E-05 | UP |
| *HSPA2* | 2.003301375 | 1.77E-05 | UP |
| *PALMD* | -2.003659089 | 0.000251865 | DOWN |
| *TYMS* | -2.010799154 | 1.36E-05 | DOWN |
| *DPYSL2* | -2.019280181 | 0.000198791 | DOWN |
| *RNASEH2A* | -2.029317006 | 5.50E-05 | DOWN |
| *CTSV* | -2.03798097 | 0.000123653 | DOWN |
| *CENPK* | -2.044548483 | 1.40E-05 | DOWN |
| *MRAP2* | -2.047650899 | 3.52E-05 | DOWN |
| *PLEKHG1* | -2.054690177 | 1.98E-05 | DOWN |
| *HERC5* | -2.076851719 | 8.59E-05 | DOWN |
| *CX3CR1* | -2.079492558 | 3.29E-05 | DOWN |
| *STAMBPL1* | -2.088573698 | 8.72E-06 | DOWN |
| *HERC6* | -2.098636361 | 1.64E-05 | DOWN |
| *SPHKAP* | -2.114115921 | 8.99E-06 | DOWN |
| *CACNA1D* | -2.118301555 | 1.78E-05 | DOWN |
| *SAMD13* | -2.11896748 | 3.69E-05 | DOWN |
| *KIF20A* | -2.168540301 | 5.76E-05 | DOWN |
| *ELAVL2* | -2.177544405 | 4.61E-05 | DOWN |
| *SELENOP* | -2.183300109 | 1.73E-05 | DOWN |
| *ZNF204P* | -2.186579233 | 5.07E-05 | DOWN |
| *GINS3* | -2.194329899 | 2.39E-05 | DOWN |
| *PRIMA1* | -2.206407583 | 3.29E-05 | DOWN |
| *CDC6* | -2.210908681 | 8.99E-06 | DOWN |
| *TTC30B* | -2.220239854 | 2.57E-05 | DOWN |
| *TMEM14A* | -2.233116405 | 8.72E-06 | DOWN |
| *CENPH* | -2.25427711 | 1.51E-05 | DOWN |
| *CENPU* | -2.25992384 | 1.18E-05 | DOWN |
| *FANCI* | -2.270635244 | 9.18E-06 | DOWN |
| *ADAMTS6* | -2.27632117 | 1.68E-05 | DOWN |
| *CMPK2* | -2.336139212 | 7.28E-05 | DOWN |
| *CALN1* | -2.338247852 | 0.000166587 | DOWN |
| *AMPH* | -2.342560804 | 3.51E-05 | DOWN |
| *FANCG* | -2.346529186 | 8.72E-06 | DOWN |
| *SEMA6D* | -2.348860596 | 1.01E-05 | DOWN |
| *PLPP3* | -2.356397426 | 1.97E-05 | DOWN |
| *TGFBR3* | -2.357064385 | 2.83E-05 | DOWN |
| *CDKN2C* | -2.381136158 | 1.71E-05 | DOWN |
| *SCN7A* | -2.397157429 | 7.81E-05 | DOWN |
| *RMI2* | -2.3985516 | 9.54E-06 | DOWN |
| *PCLAF* | -2.413337474 | 8.99E-06 | DOWN |
| *TNFAIP8* | -2.452988738 | 8.03E-06 | DOWN |
| *GINS2* | -2.472494765 | 8.72E-06 | DOWN |
| *DTL* | -2.474070635 | 9.95E-06 | DOWN |
| *TIMELESS* | -2.491315118 | 7.55E-06 | DOWN |
| *ICAM4* | -2.495481435 | 1.71E-05 | DOWN |
| *MCM5* | -2.531654512 | 1.18E-05 | DOWN |
| *MCM2* | -2.542739298 | 8.72E-06 | DOWN |
| *MCM7* | -2.544509362 | 8.72E-06 | DOWN |
| *MCM6* | -2.554195874 | 5.27E-06 | DOWN |
| *BRIP1* | -2.565857643 | 0.00014271 | DOWN |
| *FAM117B* | -2.580046882 | 1.71E-05 | DOWN |
| *ABAT* | -2.588603739 | 1.36E-05 | DOWN |
| *CCNE2* | -2.597111023 | 1.87E-05 | DOWN |
| *SYNE2* | -2.614050051 | 3.45E-05 | DOWN |
| *P2RY14* | -2.651391014 | 5.87E-05 | DOWN |
| *SFRP2* | -2.671229563 | 2.57E-05 | DOWN |
| *POLE2* | -2.738235692 | 3.88E-05 | DOWN |
| *UHRF1* | -2.786174125 | 8.03E-06 | DOWN |
| *METTL7A* | -2.852711298 | 0.000104407 | DOWN |
| *RRM2* | -2.937392681 | 8.45E-06 | DOWN |
| *PLSCR4* | -2.949355241 | 1.43E-05 | DOWN |
| *CT75* | -2.976302069 | 4.77E-06 | DOWN |
| *HELLS* | -3.285285662 | 4.44E-05 | DOWN |
| *IFIT1* | -3.332933965 | 5.22E-05 | DOWN |
| *DRD1* | -3.408379416 | 6.59E-06 | DOWN |
