# Supplementary material for: Integrated Analysis of circRNA-miRNA-mRNA ceRNA Network in Cardiac Hypertrophy
Source: Front Genet. 2022 Feb 8;13:781676. doi: 10.3389/fgene.2022.781676 (PMC8860901; doi:10.3389/fgene.2022.781676)
Supplement: Supplementary file 5 [file Table4.DOCX]

**Table S4: The circRNAs with the top 3 connectivity in two network**

| Network | Degree | CircRNA name |
| --- | --- | --- |
|  |  |  |
| Positive regulatory network | 6 | hsa_circ_0002702 |
|  | 5 | hsa_circ_0052212 |
|  | 4 | hsa_circ_0000039，hsa_circ_0025932，hsa_circ_0047301，hsa_circ_0065172，hsa_circ_0065174，hsa_circ_0074828，hsa_circ_0088188，hsa_circ_0093354，hsa_circ_0097680，hsa_circ_0123431，hsa_circ_0125882 |
| Negative  regulatory  network | 15 | hsa_circ_0047959 |
|  | 8 | hsa_circ_0013751，hsa_circ_0015164，hsa_circ_0110609 |
|  | 7 | hsa_circ_0008068，hsa_circ_0031517，hsa_circ_0061878，hsa_circ_0094964，hsa_circ_0126765 |
